# MicroRNA: A Bridge from *H. pylori* Infection to Gastritis and Gastric Cancer Development

**DOI:** 10.3389/fgene.2012.00294

**Published:** 2012-12-14

**Authors:** Jovanny Zabaleta

**Affiliations:** ^1^Department of Pediatrics, Louisiana State University Health Sciences CenterNew Orleans, LA, USA; ^2^Stanley S. Scott Cancer Center, Louisiana State University Health Sciences CenterNew Orleans, LA, USA

**Keywords:** microRNA, *Helicobacter pylori*, gastric cancer, inflammation, gastritis

## Abstract

*Helicobacter pylori* (*H. pylori*) infection is a recognized risk factor for gastric cancer. The disease is one of the most common in the world and explains for a significant number of cancer cases and cancer-associated deaths worldwide. *H. pylori* infection induces huge array of responses at the gastric epithelial cells and the immune system, inducing both pro- and anti-inflammatory molecules that are intended to either perpetuate or control the infection. Despite the strong immune response, the infection is not cleared and can persist mostly without causing major significant discomfort in the human host. Among the mediators induced in response to the infection, microRNA (miRNA) have the potential to play a major impact on the outcome of the bacteria-host interaction. These miRNA are small 18–24 nucleotide long nucleotide molecules that can interact with mRNA molecules and block their translation into proteins or induce their degradation. Many efforts have been put into the generation of miRNA profiles and their role in gastric cancer. This has led to the identification of miRNA associated with promoting the inflammatory response initiated by the *H. pylori* infection, increasing the malignant progression of the gastric epithelium, and enhancing the invasiveness and migratory capacity of cancer cells. However, at the same time, several miRNA have been associated with events that are totally opposite, leading to reduced inflammation, inhibition of malignancy and increased apoptosis of transformed cells. In summary, as it is in many other examples, the role played by miRNA in gastric cancer is the results of a delicate balance between pro- and anti-cancer miRNA, and this balance is modified by the interaction of many players, many of which are still waiting to be discovered.

## Introduction

According to the American Cancer Society (ACS), cancer is the second most common death cause in the United States^1^. Even though the survival rate (up to 5-years) has improved tremendously over the last decades, it is expected that more that 1.5 million Americans will die to cancer in 2012 (Siegel et al., 2012). The incidence and mortality of gastric cancer has followed that of most cancers but still more than 10,000 deaths are expected to happened in 2012 (1.3% of all cancer cases and 7.5% of digestive system cancers; Siegel et al., 2012). According to the Surveillance Epidemiology and End Results (SEER)^2^, gastric cancer is a disease of disparities, in terms of incidence and mortality, with African American men and women having the highest incidence and mortality rates of gastric cancer when compared to other races/ethnic groups. In addition, males from all ethnicities have higher incidence and mortality rates than females (see text footnote 2). There are several theories to explain these differences and based on several facts, it seems that estrogens are important modulators of gastric cancer risk (Camargo et al., 2012). Additional disparities in gastric cancer incidence and mortality rates are observed by geographic region with Japan and China having more than 20 cases of gastric cancer and North America presenting less than 10 cases per 100,000 individuals (Parkin, 2004; Parkin et al., 2005). All these observations led to the idea that environmental factors, including diet, play a major role in gastric cancer risk. However, recent findings have suggested a significant role of the genetic background in gastric cancer susceptibility. There have been plenty of publications showing association of single nucleotide polymorphisms (SNP) and risk of gastric cancer. We have shown also that these SNPs may serve as biomarkers of risk even at earlier stages, during the progression of inflammatory to malignant gastric stages (Zabaleta et al., 2006, 2008; Zabaleta, 2012). Identifying early markers of risk of GC is crucial because the disease has one of the highest rates of mortality worldwide. However, this task has been complicated by several factors, including difficulties for tissue collection (which is generally obtained by gastric biopsies); the presence of millions of SNPs in the genome and the lack of studies determining the degree of interactions among them; the time-dependent expression of genes which leads to misinterpretation of gene profiles; and very especially, the lack of studies validating genomic profiles generated from tissue samples in more easily obtained samples, like blood or urine, making replication of results less invasive and less hazardous and stressful for the patient.

## microRNA

Since its discovery, microRNA (miRNA) added a new chapter in the study of gene regulation. It was initially observed that the expression of the heterochronic protein, those that control temporal development, Lin-14 during the development of *Caenorhabditis elegans* (*C. elegans*) lead to a temporal expression of several cell lineages (Ruvkun and Giusto, 1989). This temporal expression of Lin-14 protein leading to different genotypes suggested a strong regulation in the gene encoding its expression, *Lin-14*. It was later discovered that the expression of Lin-14 protein was down-regulated at the post-transcriptional level by two products of the gene *Lin-4*, another heterochronic gene, whose products bind to the untranslated (UTR) 3′ region of the *Lin-14* gene (Wightman et al., 1993). In an interesting series of papers published in the same issue of Science Magazine, three back-to-back papers showed that these small RNA molecules were present in organisms other than *C. elegans*, and the authors coined the term miRNA for them (Lagos-Quintana et al., 2001; Lau et al., 2001; Lee and Ambros, 2001). These miRNA result from the activity of two RNase III, Drosha, and Dicer, that process the primary miRNA into mature miRNA of 18–24 nucleotides that mediate inhibition of translation or degradation of messenger RNA (mRNA; Bartel, 2004) by base mispairing with mRNA (RNAi; Olsen and Ambros, 1999), giving the miRNAs a broad potential to regulate gene expression (Bartel and Chen, 2004). However, the degree of specificity for the binding of the miRNA with its target mRNA seems to be given by the first nine 5′ nucleotides (seed nucleotides) of the miRNA and their complementary 3′ untranslated regions (3′UTR) in their targets (Moss et al., 1997; Reinhart et al., 2000; Kiriakidou et al., 2004; Vella et al., 2004).

## miRNA Nomenclature

The criteria for the identification of nucleotide sequences as miRNA were consented by a group of experts on the topic (Ambros et al., 2003). These researchers delineated certain characteristics that, except for some specific conditions, should be met by the sequence to be considered a miRNA, including the detection of a ∼22 nt RNA molecule, identification of that molecule on a pool of cDNA made from RNA with specified sizes, the presence of a hairpin, phylogenetic conservation of the molecule and its precursor, and increased expression of the precursor RNA molecules in the absence of Dicer (Ambros et al., 2003). miRNAs are named based on several criteria, including: three or four letters to designate species (e.g., hsa for *Homo sapiens*); mature miRNAs are given the designation of “miR” while precursor sequences are named “mir”; sequential numbers; miRNAs that differ in only one or two nucleotides are given letter suffixes, i.e., mmu-miR-10a and mmu-miR-10b; if two different miRNAs are generated from the opposite arms of the hairpin are named with an additional suffix indicating the 5′ or the 3′ where the miRNA is generated from, i.e., hsa-miR-140-5p and hsa-miR-140-3p; in addition, when two miRNAs originate from opposite arms of the hairpin, the one with reduced expression is annotated with an asterisk (*; Ambros et al., 2003; Griffiths-Jones, 2005; Griffiths-Jones et al., 2006, 2008).

## miRNA and *Helicobacter pylori* Infection

Among several factors, infection with *Helicobacter pylori* (*H. pylori*) pylori is considered to be a crucial event associated with risk of gastric cancer. Such is the importance of the infection in the inflammation that leads to gastric cancer that *H. pylori* has been classified as a Type I carcinogen by the International Agency for Research in Cancer (IARC; IARC, 1994). After infection to the gastric mucosa, *H. pylori* injects the product of the cytotoxin-associated gene (cag; CagA) into the gastric epithelial cells by a type IV secretion system (Backert et al., 2000; Odenbreit et al., 2000). Once there, CagA is phosphorylated (Stein et al., 2002) and induces a cascade of kinases activation leading to cellular changes (Higashi et al., 2002) and production of inflammatory cytokines (Orsini et al., 2000; Lai et al., 2011). Interestingly, using an *in vitro* system, it was shown that CagA induces hsa-miR-584 and hsa-miR-1290 in a NFκβ and Erk 1/2 dependent manner, respectively (Zhu et al., 2012). Another set of experiments has shown that after *H. pylori* infection there is a strong induction of hsa-miR-155 which inhibits the production of the potent pro-inflammatory cytokine IL8, through the inhibition of the NFκβ pathway by interacting with the 3′-UTR of the Iκβ kinase (Xiao et al., 2009; reviewed in Ma et al., 2011). Interestingly, in addition to hsa-miR-155, *H. pylori* infection also induced hsa-miR-146a (Xiao et al., 2009). This miRNA, in addition to hsa-miR-155 and hsa-miR-132, is produced in response to inflammatory stimulus like LPS (Taganov et al., 2006). Interestingly, similar to hsa-miR-155, miR-146 also regulates the NFκβ pathway by targeting *TRAF6* and *IRAK1* (Taganov et al., 2006; Liu et al., 2010). Through the regulation of *TRAF6* and *IRAK1*, hsa-miR-146a modulates the inflammatory response induced by *H. pylori* by reducing the levels of IL8, MIP-3α, and GRO-α (Liu et al., 2010), suggesting that this miRNA plays an essential role in the control of the inflammatory response to *H. pylori* and possibly in the limitation of tissue damage observed in patients with gastritis and gastric cancer. In addition, hsa-miR-146a also regulates the expression of the *PTGS2* gene (Liu et al., 2012b), which produces prostaglandin E2 that has been associated with *H. pylori* infection and concomitant infiltration of inflammatory cells to the gastric mucosa (Fu et al., 1999; McCarthy et al., 1999). As a quick reference, the association of several miRNA with *H. pylori* infection, gastritis, and gastric cancer is shown in a summarized way in Table 1.

**Table 1:** **Association of several miRNA with gastric lesions, from *H. pylori* infection to gastric cancer**.

hsa-miR-#	Observation	Reference
9, 146a, 155, 650, 96, 204	Chronic active gastritis (NAG)	Lario et al. (2012)
21, 223, 218, 25	*H. pylori* infection, gastric cancer	Li et al. (2012a)
155, 146a	Modulation of IL8, MIP3a, GRO-a, Reduced PTGS2 in *H. pylori* infection	Liu et al. (2010), Liu et al. (2012b), Xiao et al. (2009)
103, 200b, 200c, 375, 532	*H. pylori*-induced gastric inflammation	Isomoto et al. (2012)
let-7	Induced by CagA, accumulation of Ras	Hayashi et al. (2012)
17, 20 21 223	Reduced in metaplastic and non-metaplastic cancerous glands after *H. pylori* eradication	Shiotani et al. (2012)
17, 204	Increased after *H. pylori* eradication	Shiotani et al. (2012)
155, 584, 1290	Effect on severity of gastritis and presence of more advanced gastric lesions	Oertli et al. (2011), Zhu et al. (2012)
150	Increased in gastric cancer tissues; induces cell migration and invasion by reducing the expression of EGR2	Wu et al. (2010)

## miRNA and Gastric Inflammatory Stages

*Helicobacter pylori* infection may last many years without inducing any type of gastric discomfort to its human host. Even though between 1 and 3% of infected people develop gastric cancer (Uemura et al., 2001; Suerbaum and Michetti, 2002; Wroblewski et al., 2010), the majority of infected individuals develop a continuous and progressive chronic inflammatory process initiated by non-atrophic gastritis and followed by multifocal atrophic gastritis, intestinal metaplasia, and dysplasia; the latter is considered the truly precancerous stage of the cascade (Correa et al., 1975; Zabaleta et al., 2006; reviewed in Zabaleta, 2012). The rate of change to more advanced gastric lesions is higher than the rate of regression (Correa et al., 1990). Even though pathological features clearly distinguish between each inflammatory stage, the molecular signatures of these transitions have not being explored and the underlying mechanisms are unknown. It has been shown that the level of the pro-inflammatory cytokines IL1β, IL6, IL8, and TNFα were positively correlated with the level of chronic gastritis but that correlation disappear in the presence of gastric atrophy and was inverse, for IL6 and IL8, in intestinal metaplasia (Isomoto et al., 2012). Interestingly, the levels of these cytokines were, in general, inversely correlated with the levels of several miRNA in the gastric mucosa. For example, the levels of miRNA let-7b were inversely correlated (−0.59) with *IL1B* levels (*p* < 0.005) while hsa-miRNA-103 correlated with *IL6* (−0.612, *p* < 0.005), hsa-miR-375 with *IL8* (−0.469; *p* < 0.05), and hsa-miR-200a with *TNFA* (−0.606; *p* < 0.005; Isomoto et al., 2012). A profile of several miRNA was associated with reduction of all inflammatory cytokines, suggesting a common mechanism for the control of the expression of these inflammatory mediators (Isomoto et al., 2012). Interestingly, hsa-miR-155 deficient mice present reduced gastritis when compared to wild type mice (Oertli et al., 2011). These responses are associated with increased numbers of *H. pylori* CFU in the stomachs of infected hsa-miR-155 deficient mice and reduced CD4+ T-cell response evidenced by low production of IFNγ and IL17 (Oertli et al., 2011). After a long follow-up, mice over expressing hsa-miR-584 and hsa-miR-1290 showed changes associated with gastric intestinal metaplasia including the appearance of colonic epithelium and colonic markers (Muc-2; Zhu et al., 2012), suggesting a role of these two miRNA in the development of more advanced gastric lesions. However, the role played by *H. pylori*, if any, was not determined in this *in vivo* follow-up. It is possible that the presence of *H. pylori* infection may shorten the time for the appearance of the epithelial abnormalities. However, even after eradication of *H. pylori* with a triple antibiotic treatment (amoxicillin, clarithromycin, and pump inhibitors) for a 7-day period, the levels of known oncogenic miRNA, including hsa-miR-21, hsa-miR-25, and hsa-miR-93, did not change (Shiotani et al., 2012). In contrast, the levels of tumor-suppressor miRNA, including let-7 and hsa-miR-204, increased after eradication (Shiotani et al., 2012). These results suggest that after infection and eradication of *H. pylori*, some underlying processes may continue that promote tissue damage and lead to gastric malignancy. In addition, it is also suggested that more than a single isolated response, the articulated and balanced reaction to the infection and to the inflammatory process dictates the outcome of the cascade initiated by the *H. pylori* infection. In addition to the inflammatory cascade associated with the development of gastric adenocarcinoma, several miRNA have been linked to *H. pylori-*induced mucosal associated lymphoid tissue (MALT) lymphomas. Using an array of 376 miRNA Thorns et al. (2012) found 41 miRNA associated with changes from normal gastric mucosa to gastritis and to MALT. Interestingly, the levels of some of these miRNA seem to change in response to the infiltration of lymphocytes or the presence of *H. pylori* while only a few (hsa-miR-150, hsa-miR-550, hsa-miR-124a, hsa-miR-518b, and hsa-miR-539) were associated with lymphoma and presented a steady increase from gastritis to MALT (Thorns et al., 2012). Taken together these results suggest that miRNA may be modulating pathways associated with differential outcomes in response to a common trigger, infection with *H. pylori*. This supports the concept about universality of the miRNA responses and opens up the possibility of more efficacious and global treatments for illnesses with common origins.

## miRNA and Gastric Cancer

It has been shown that the distribution of miRNAs on the human genome is non-random but rather, a significant fraction of them are located on chromosomal regions known as “fragile sites” and on genomic regions associated with cancer (Calin et al., 2004). In fact, according to the same report, the incidence of miRNA in these fragile sites is more than ninefold higher than in “non-fragile” regions (Calin et al., 2004). Due to their ability to interact with mRNA, a single miRNA can act as either tumor-suppressors or oncogenes, meaning that a miRNA can be classified as tumor-suppressor- or onco-miRNA, depending on the context of their expression, as has been shown for the miRNA cluster miR-17-92 (He et al., 2005; O’Donnell et al., 2005). Over expression of onco-miRNAs may target certain tumor-suppressor genes and allow the activity of oncogenes and their targets. On the contrary, over expression of tumor-suppressor miRNA would limit the transcription of genes associated with tumorigenesis, cell division, migration and invasion, and metastasis. In this sense, for example, it has been shown that hsa-miR-135a promotes metastasis in breast cancer cell lines by direct interaction with *HOXA10* gene, which acts as a metastasis suppressor in this cancer model (Chu et al., 2004; Chen et al., 2012). Due to the dual role of miRNA in cancer and to the dynamic nature of these gastric lesions, it would be recommended to establish the association with disease using several miRNA, or miRNA profiles, rather than doing it with individual miRNA. An early miRNA profile in gastric cancer showed that stomach tissues have a specific miRNA signature that is similar to that of pancreas, colon, and prostate cancers but different from that of breast and lung cancer (Volinia et al., 2006).

The association of specific miRNA or miRNA signatures with gastric cancer is at several levels. One very frequent feature found is that miRNA modify the capacity of gastric cancer cells to proliferate, migrate, and invade. Using *in vitro* and *in vivo* systems several authors have shown that miRNAs act as inductors of cell proliferation and inhibitors of apoptosis by interacting with and inhibiting the expression of known tumor-suppressor genes. Wu et al. (2010) showed that overexpression of hsa-miR-150 increases the proliferation of gastric cell lines, as compared to non-treated cells or cells treated with hsa-miR-150 antagonists. hsa-miR-150 transfected cell lines were also able to induce tumor formation in nude mice. Interestingly, hsa-miR-150 was increased in gastric cancer cell lines as well as in gastric cancer tissues, while its expression was reduced in normal adjacent and distant tissues (Wu et al., 2010). Later analysis showed that the effect of hsa-miR-150 was associated with reduced expression of the gene *EGR2* (Wu et al., 2010), which has been described as a tumor-suppressor (Unoki and Nakamura, 2001). The *EGR2* gene seems to be one of the mediators of the antitumor effects of PTEN (Matsushima-Nishiu et al., 2001; Unoki and Nakamura, 2001), which in turns, is reduced by the interaction with another miRNA, hsa-miR-21 (Zhang et al., 2012a). On the other hand, Zhang et al. (2012b) have described an increased expression of hsa-miR-181a in gastric tumors, as compared to normal tissues. This miRNA interacts with the 3′-UTR of the tumor-suppressor gene *KLF*, which is reduced in the same gastric tissues where miR-181a was measured, and is able to inhibit apoptosis of gastric cell lines (Zhang et al., 2012b). A recent publication (Zhu et al., 2010) shows that miRNA let-7a expression is significantly reduced in gastric epithelial cancer cells when compared to normal cells, especially in samples from patients with lymph node metastasis.

After being able to maintain certain level of cellular proliferation, cancer cells must gain the capacity to cross the basal cellular layers of the epithelia and get into the blood stream to metastasize to local lymph nodes and farther tissues. In two different approaches researchers have shown an important role for the gene inhibitor of growth (*ING4*) which is targeted by hsa-miR-650 and hsa-miR-622 and this in turn, seems to be associated with increased invasion/migration of gastric cell lines *in vitro* and promote metastasis in nude mice (Zhang et al., 2010; Guo et al., 2011). In synthesis, the success of a gastric cancer cell to invade and become metastatic depends on the ability of that cell to promote or down-regulate the expression of proteins involved in cell cycle, adhesion, cell-to-cell contact, among other things. The balance between miRNA with activities pro- and anti-tumorigenesis may in part determine the final outcome and define the fate of the tumor. So, there are miRNA profiles that promote tumor growth including hsa-miR-622, hsa-miR-650, hsa-miR-223, hsa-miR-21, and hsa-miR-181a, among others (Zhang et al., 2010, 2012a,b; Guo et al., 2011; Li et al., 2011b); while hsa-miR-107, -145, -495, -551a, let-7f, -218, and -610, among others, inhibit cell invasion and metastasis (Tie et al., 2010; Li et al., 2011a, 2012b; Liang et al., 2011; Feng et al., 2012; Gao et al., 2012; Wang et al., 2012).

## miRNA and Its Role as Biomarkers and Predictors of Gastric Cancer

Compared with the much higher number of mRNA, about 1,000 miRNA have been validated in humans, making feasible the generation of genetic profiles, with microarrays and high throughput sequencing, to associate with disease and disease outcome, or to identify possible biomarkers. In addition, due to its size, miRNA are very stable in biological fluids facilitating the profiling with non-invasive methods. After creating miRNA profiles in gastric cancer, colorectal cancer, and healthy controls, 7 miRNA were identified as specific for gastric cancer (Liu et al., 2012a). However, after validating these 7 miRNA in an independent set of samples, miR-187*, miR-371-5p, and miR-378 remained significantly associated with gastric cancer serum samples (Liu et al., 2012a). Further analysis revealed that hsa-miR-378 had a better biomarker potential and this was corroborated by showing that the level of hsa-miR-378 started to increase very early during the cancer development making it a possible early detection marker for gastric cancer (Liu et al., 2012a). Additional studies have identified several other miRNA that could serve as biomarkers and predictors of gastric cancer (Liu et al., 2011; Li et al., 2012a; Song et al., 2012). The differences observed in the profiles may be related to several things, including ancestry, density of the platform used for the profiling (i.e., microarray, TaqMan arrays), which may allow for lower or higher input of sequences and therefore, limit the probability of identifying specific miRNA. Whatever the technology used, it is clear that miRNA profiling is a potent tool that can be used to improve both diagnosis and prognosis in gastric cancer. The current knowledge of the miRNA role in the pathogenesis of gastric cancer make these as potential targets to either improve therapeutic options currently in use, or to devise new strategies for the treatment of the disease. These treatments have to be directed to at least two things, to reduce the malignant process that lead to hyper-proliferation of gastric cells and are associated to malignant transformation, and to reduce the inflammatory response that promote the influx of immune cells into the gastric mucosa. In addition, it is very possible that *H. pylori* has its own set of miRNA that can affect the immune response of the host in order to increase the chances of perpetuating its infection.

## Conclusion

microRNA profiles have been established, and most probably will be used as molecular targets to modify the interaction of *H. pylori* with gastric cells and to reduce the inflammation and cellular malignancy that may lead to gastric cancer. Among profiles of mRNA, methylation (cpG), genome wide association studies (GWAS), miRNA profiles have the highest potential to successfully become widely used to reach the goal of “personalized medicine” by which, a patient is to be medically treated according to his/her genetic profile. These profiles, it is expected, would classify and predict the outcome of a disease better than traditional techniques, as well as dictate the steps to follow in order to better treat that specific patient.

## Conflict of Interest Statement

The author declares that the research was conducted in the absence of any commercial or financial relationships that could be construed as a potential conflict of interest.

## Footnotes

^1^http://www.cancer.org

^2^http://seer.cancer.gov/statfacts/html/stomach.html
